# Smartphone Cardiac Rehabilitation, Assisted Self-Management (SCRAM) Versus Usual Care: Multicenter Randomized Controlled Trial

**DOI:** 10.2196/66074

**Published:** 2026-03-17

**Authors:** Ralph Maddison, Narayan Subedi, Peixuan Li, Karen E Lamb, Kylie Ball, Brian Oldenburg, Clara Chow, Sarah A McNaughton, Elena S George, Lan Gao, Marj Moodie, John Amerena, Voltaire Nadurata, Christopher Neil, Stuart Cameron, Jonathan C Rawstorn

**Affiliations:** 1 Institiute for Physical Activity and Nutrition, School of Exercise and Nutriton Sciences, Deakin University, 221 Burwood Highway, Burwood, Melbourne, Victoria, 3125, Australia, 61 3 924 46218; 2 Centre for Epidemiology and Biostatistics, Melbourne School of Population and Global Health, The University of Melbourne, Melbourne, Victoria, Australia; 3 Methods and Implementation Support for Clinical and Heath Research Hub, Faculty of Medicine, Dentistry and Health Sciences, The University of Melbourne, Melbourne, Victoria, Australia; 4 Baker Heart and Diabetes Institute, Melbourne, Victoria, Australia; 5 Department of Cardiovascular Research, Translation and Implementation, School of Psychology and Public Health, La Trobe University, Melbourne, Victoria, Australia; 6 Westmead Applied Research Centre, Faculty of Medicine and Health, The University of Sydney, Sydney, New South Wales, Australia; 7 School of Human Movement and Nutrition Sciences, The University of Queensland, Brisbane, Queensland, Australia; 8 Deakin Health Economics, Institute for Health Transformation, Deakin University, Melbourne, Victoria, Australia; 9 Geelong Cardiology Research Unit, Barwon Health, Geelong, Victoria, Australia; 10 Department of Cardiology, Bendigo Health, Bendigo, Victoria, Australia; 11 Heart Wise Cardiology, Melbourne, Victoria, Australia; 12 Applied Artificial Intelligence Institute, Deakin University, Melbourne, Victoria, Australia

**Keywords:** mHealth, cardiac rehabilitation, telerehabilitation, rural, coronary heart disease, randomized controlled trial

## Abstract

**Background:**

Accessibility barriers contribute to low participation in center-based cardiac rehabilitation. We developed an innovative, comprehensive, dual-phase telerehabilitation program to address this gap (Smartphone Cardiac Rehabilitation, Assisted Self-Management; SCRAM).

**Objective:**

The study aimed to determine the effectiveness of SCRAM for increasing maximal aerobic exercise capacity (VO_2_max).

**Methods:**

A multicenter, parallel 2-arm randomized controlled trial recruited clinically stable adults (aged ≥18 y) with diagnosed coronary heart disease at 3 hospitals in Victoria, Australia (Melbourne, Geelong, and Bendigo) from 2018 to 2021. Participants were randomized (1:1), stratified by sex and study site, to receive SCRAM plus usual cardiovascular care (intervention) or usual cardiovascular care alone (control). SCRAM provided 24 weeks of remote exercise supervision, coaching, and behavior change support via smartphone. Usual cardiovascular care included standard medical care and advice to seek a referral to center-based cardiac rehabilitation, which was heavily impacted during the COVID-19 pandemic. Due to the nature of the treatments, participants were not blinded to allocation; primary outcome assessors and biostatisticians were blinded. The primary outcome was VO_2_max at 24 weeks, analyzed on the principle of intention-to-treat, using linear regression adjusted for baseline and stratification factors on multiple imputed data.

**Results:**

Recruitment and data collection were heavily impacted by COVID-19, although SCRAM delivery was sustained throughout. Of 220 required participants, only 123 (56%) were recruited and randomized (intervention n=63, control n=60); 45% (55/123) had missing VO_2_max at 24 weeks—largely due to enforced COVID-19 restrictions. Mean VO_2_max at 24 weeks favored SCRAM (26.10, SD 10.72 mL/kg/min) over control (24.65, SD 7.87 mL/kg/min), but the difference was not statistically significant (mean difference=1.61 mL/kg/min, 95% CI –1.38 to 4.61, *P*=.28). Among secondary outcomes, patients receiving SCRAM had lower diastolic blood pressure at 24 weeks (mean difference=–5.54 mm Hg, 95% CI –10.01 to –1.06). All reported adverse events (control n=6, intervention n=16) were deemed mild or moderate, with only one deemed as possibly related to treatment. There were no deaths or hospitalizations.

**Conclusions:**

This was an underpowered trial, but SCRAM did not lead to a clinically important difference in VO_2_max compared to usual cardiac care. SCRAM was resilient to COVID-19–related disruptions that significantly impacted the delivery of cardiac rehabilitation and supervised exercise training in particular. Further research is needed to conclusively assess treatment effects and understand how virtual cardiac rehabilitation can be translated into routine practice to augment center-based delivery and enhance equity of access.

## Introduction

Cardiovascular disease (CVD) is the leading cause of death and disease burden, globally [1]. Since 1990, the burden of CVD has continued to increase in most countries, with trends driven by changing exposures to harmful risk factors, population growth, and population aging [1]. For people living with CVD, secondary prevention comprising lifestyle modifications, pharmacotherapy, and cardiac rehabilitation is recommended to reduce the risk of recurrent events and is a priority of the World Heart Federation [2].

Cardiac rehabilitation has a class 1a recommendation for management of CVD, particularly for coronary heart disease (CHD) [3-5]. Cardiac rehabilitation programs deliver comprehensive support, education, and monitoring of patients after a cardiovascular event. In a meta-analysis of 85 randomized controlled trials (RCTs) involving 23,430 participants with CVD, exercise-based rehabilitation was associated with significant risk reductions in cardiovascular mortality (risk ratio [RR]: 0.74, 95% CI 0.64‐0.86, hospitalizations [RR: 0.77, 95% CI 0.67‐0.89], and myocardial infarction [RR: 0.82, 95% CI 0.70‐0.96]). There was evidence that cardiac rehabilitation improved health-related quality of life (HRQoL) and was cost-effective [6].

However, low participation rates limit the benefits of traditional center-based (face-to-face) programs [7-11]. Literature shows that 50%‐70% of patients eligible for cardiac rehabilitation do not attend, and among those that do attend, 30%‐60% do not complete their program [12,13]. Barriers to use are diverse and include distance to services and time pressures caused by the need for face-to-face in-hospital or clinical-setting attendance [14]. Evidence-based alternative delivery models are needed to overcome access barriers and improve participation [15,16].

Home-based delivery models have been designed to overcome aforementioned barriers to center-based cardiac rehabilitation. A Cochrane systematic review and meta-analysis of 24 RCTs involving 3046 participants that compared center- and home-based rehabilitation programs reported similar effectiveness for improving clinical, functional, and patient-reported outcomes, and low risk of adverse events [17]. These findings are encouraging, but they also identified a lack of interaction between participants and rehabilitation professionals during home-based delivery.

A small number of trials included in the Cochrane review augmented home-based delivery with digital technologies. Often known as telerehabilitation, this approach has rapidly gained interest because it can support interaction between participants and rehabilitation professionals and enable important program components such as social support and personalization. Indeed, the European Society of Cardiology recently defined telerehabilitation as a key quality indicator for cardiac rehabilitation program accreditation and undertook a Delphi methodology to identify minimum standards for high-quality delivery [18]. Recommendations highlight the potential benefits of both synchronous and asynchronous remote monitoring of exercise training. These approaches have also been outlined by the American Heart Association [19]. Synchronous monitoring involves viewing exercise data live during training sessions and could enable higher levels of responsiveness, personalization, and feedback but is more resource-intensive to deliver. Asynchronous monitoring involves intermittently reviewing previously recorded exercise data and could provide more flexibility for participants and professionals and promote greater autonomy for reducing resource use but offers less interaction and support from rehabilitation professionals [18,20].

These attributes may make synchronous monitoring well-suited during earlier program stages (eg, phase II) and for individuals whose medical and exercise history indicates a need for closer supervision. Conversely, asynchronous monitoring may be well suited for later program stages (eg, phase III and beyond) and for individuals whose medical and exercise history is suitable for more self-directed exercise. These approaches could be combined sequentially to provide a graduated program of long-term support that emphasizes safety and personalization on entry before transitioning toward self-direction and lifelong behavior change.

We previously developed and evaluated the 12-week REMOTE-CR (remote exercise monitoring trial for exercise-based cardiac rehabilitation) telerehabilitation program, which delivered synchronous remote exercise coaching via smartphone and wearable sensing technologies [21,22]. The effect on maximal aerobic exercise capacity (VO_2_max; primary outcome) was noninferior to center-based programs (adjusted mean difference −0.51, 95% CI −0.97 to −1.98 mL/kg/min; *P*=.48; prespecified inferiority margin: −1.25) [23]. REMOTE-CR was also substantially cheaper to deliver and had high usability, acceptability, and end-user demand [23,24].

Following the REMOTE-CR trial, we extended the intervention to include asynchronous exercise and behavioral support for an additional 12 weeks (total 24 wk) and to incorporate evidence-based multifactorial support for self-management behaviors [25] (eg, diet, medication adherence). The extended intervention, named Smartphone Cardiac Rehabilitation, Assisted Self-Management (SCRAM) comprised additional modular components in the form of push notifications that were derived from our previous research studies [26-28] and expert input.

The aims of this study were to compare the effects of the SCRAM intervention on exercise capacity, lifestyle and self-management behaviors, and HRQoL with usual cardiovascular care in adults with CHD.

We hypothesized that SCRAM would augment usual secondary prevention services and improve cardiorespiratory fitness compared to controls. In addition, we hypothesized that SCRAM would have a positive effect on other lifestyle behaviors including diet and physical activity.

## Methods

### Trial Design

A pragmatic multicenter 2-arm parallel-group RCT was conducted between November 2018 and August 2021. Participant eligibility criteria were unchanged after trial commencement, but enforced COVID-19 restrictions affected recruitment, outcome measurement, and access to usual cardiovascular care. The trial protocol was prospectively registered (ACTRN12618001458224, August 30, 2018), published [29], and reported according to CONSORT (Consolidated Standards of Reporting Trials) guidelines (Checklist 1) [30]. Analyses were outlined in a detailed plan prior to unblinding (Multimedia Appendix 1) with postunmasking changes noted.

### Changes to Trial Protocol

Enforced responses to COVID-19 at study sites required a change in the way the primary outcome was measured for some participants (see Outcomes section), and changed delivery of the control treatment (see Control section). Difficulty achieving the target sample size also resulted in changes to the preplanned analyses (Multimedia Appendix 1).

### Eligibility and Recruitment

Clinically stable (no hospitalization within 6 wk) adults (≥18 y) with a recent CHD diagnosis (angina, myocardial infarction, and coronary revascularization within 6 mo) were recruited from 3 hospitals (Sunshine Hospital, Western Health; University Hospital Geelong, Barwon Health; Bendigo Hospital, Bendigo Health) in Victoria, Australia (November 12, 2018 to March 21, 2021). Research nurses identified participants from in-patient records and outpatient clinics and provided verbal and written information. Consenting participants were scheduled for baseline assessment.

### Intervention

**Figure 1.**
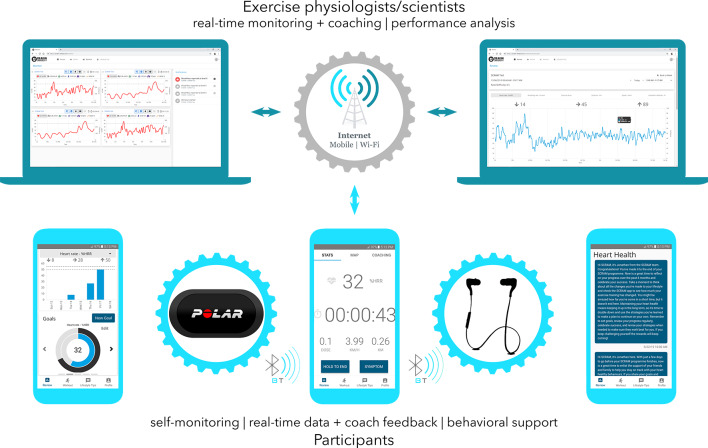
Schematic diagram of the Smartphone Cardiac Rehabilitation, Assisted Self-Management platform.

Participants received SCRAM plus usual cardiovascular care. SCRAM has been described fully elsewhere [29], according to recommendations in the Template for Intervention Description and Replication [31]. Briefly, SCRAM was a multicomponent dual-phase intervention that provided participants with a comprehensive, 24-week individualized, evidence-based program of exercise training and modular behavioral self-management support via a bespoke smartphone platform. The SCRAM platform included a Polar wearable sensor, a participant-facing app that is compatible with iOS and Android (≥v5.0) operating systems, and a health professional-facing web app, compatible with mobile and desktop web browsers (Figure 1). Behavior change strategies and education were delivered via staged modular push notifications to support the uptake and maintenance of healthy eating, physical activity, medication taking, stress management, and, if indicated, smoking cessation. Each module provided 2‐4 notifications per week in weeks 1‐12 and 1‐3 notifications per week in weeks 13‐24. As highlighted above, the additional modules were derived from our previous trials [26-28] as well as expert input from an exercise physiologist (JCR) and dietitian (ESG) to ensure relevance for the Australian context. Participants accessed the smartphone app via Apple and Google Play Stores.

During weeks 1‐12, accredited exercise physiologists provided synchronous remote exercise prescription, monitoring, and coaching via the SCRAM platform. Weeks 13‐24 transitioned participants toward independent, self-determined exercise and behavior change. Exercise physiologists provided weekly coaching phone calls informed by asynchronous review of exercise data, rather than synchronous supervision.

### Control

Participants received usual cardiovascular care, including advice to seek referral to center-based cardiac rehabilitation.

### Outcomes

#### Primary Outcome

Maximal oxygen uptake (VO_2_max, mL/kg/min) was assessed at baseline and 24 weeks during an incremental treadmill test. When direct measurement (respiratory gas analysis) was precluded by COVID-19 restrictions, VO_2_max was estimated from treadmill velocity and gradient using established metabolic equations for treadmill walking (<8 km/h: [velocity {m/min} * 0.1] + [velocity {m/min} * gradient {%} * 1.8]+3.5) or running (>8 km/h: [velocity {m/min} * 0.2] + [velocity {m/min} * gradient {%} * 0.9]+3.5) [32].

#### Secondary Outcomes

Secondary outcomes are summarized in Table 1. COVID-19 restrictions prevented objective outcome measurement for some participants. Self-reported physical activity was assessed using the Godin Leisure-time Physical Activity Questionnaire [33]. Dietary intake was assessed using the web-based, Automated Self-Administered 24-Hour Dietary Assessment Tool [34-37]. Participants completed the Automated Self-Administered 24-Hour Dietary Assessment Tool for 1 calendar weekday within 3 days of assessment appointments. Alcohol consumption was assessed using the Alcohol Use Disorders Identification Test-C [38], while the Medication Adherence Scale [39] assessed self-reported medication adherence. HRQoL was assessed using the Assessment of Quality of Life 8-Dimension scale [40]. The 8 dimensions include independent living, happiness, mental health, coping, relationships, self-worth, pain, and senses. Methods for prespecified health economic analyses have been reported in detail elsewhere [41], and the results will also be reported separately. We carried out semistructured exit interviews with trial participants, which were categorized under the themes of usability, acceptability, and satisfaction. Process evaluation outcomes using the Reach, Effectiveness, Adoption, Implementation, and Maintenance framework will also be reported as a separate paper.

**Table 1.** Secondary outcomes^a^.

Outcome	Baseline	Wk 12	Wk 24
Objective			
BP^b^ systolic or diastolic (mm Hg)	✓	N/A^c^	✓
Body mass (kg)	✓	N/A	✓
BMI (kg/m^2^)	✓	N/A	✓
Waist or hip circumference (cm)	✓	N/A	✓
Waist hip ratio	✓	N/A	✓
Fasted blood lipids (mmol/L)	✓	N/A	✓
Fasted blood glucose (mmol/L)	✓	N/A	✓
Subjective			
Physical activity (% reporting Godin LSI^d^ ≥14 units) [33]	✓	✓	✓
Medication adherence (% reporting score=4) [39]	✓	✓	✓
Alcohol consumption (% reporting ≤2 drinks/d) [38]	✓	✓	✓
HRQoL^e^ (AQoL-8D^f^, multiattribute utility score) [40]	✓	✓	✓
Vegetable intake (servings/d) via ASA24-Australia^g^ [34-37].	✓	✓	✓

a Randomized controlled trial, Victoria, Australia. Population: adults with coronary heart disease.

b BP: blood pressure.

c NA: not assessed.

d LSI: leisure score index.

e HRQoL: health-related quality of life.

f AQoL-8D: Assessment of Quality of Life-8 Dimensions.

g ASA24-Australia: Automated Self-Administered 24-Hour Dietary Assessment Tool.

Adverse events were reported at 12 and 24 weeks and classified for severity (mild, moderate, and severe) and likely relationship to study treatment (not related, possible, probable, and definite).

### Sample Size

A total of 220 participants were required to detect a clinically important 2 (SD 6.75) mL/kg/min between-group difference in VO_2_max at 24 weeks with 2-sided α of .05, 90% power, assuming preintervention and postintervention correlation of 0.8 [42], and 20% attrition [29].

### Randomization

An independent biostatistician created a computer-generated randomization schedule, stratified by sex and trial site. Other researchers did not have access to the sequence. Participants were allocated to receive intervention or control treatments at a 1:1 ratio using a centralized web-based system (Research Electronic Data Capture [REDCap]; Vanderbilt University) that ensured allocation concealment until the time of randomization.

### Blinding

Due to the nature of the intervention, participants could not be blinded to allocation. Primary outcome assessors and biostatisticians were blinded to allocation. Secondary outcomes were either self-reported or assessed by research nurses or researchers who were not blinded to allocation.

### Statistical Analysis

Available data were analyzed according to randomized assignment (ie, intention-to-treat). A linear regression model was fitted to estimate the absolute mean between-group difference in VO_2_max at 24 weeks, irrespective of participant adherence, adjusting for baseline VO_2_max and stratification factors (sex and study site). Similar analyses were undertaken for continuous secondary outcomes, except for HRQoL. A linear mixed model was fitted for HRQoL accounting for treatment arm, time, treatment arm by time interaction, baseline value, and stratification factors. Logistic regression models with generalized estimating equations were fitted for binary secondary outcomes (physical activity, alcohol consumption, and medication adherence), and an ordinal regression model with clustered standard errors was fitted for vegetable consumption; these models accounted for treatment arm, time, treatment arm by time interaction, baseline values of each outcome and stratification factors, sex, and study site.

Primary analysis used multiple imputations due to high levels of missing follow-up data. Complete case analyses were undertaken in sensitivity analyses. Per protocol analyses excluded participants with major protocol violations or nonadherence to the SCRAM intervention (recording <12 exercise sessions via the SCRAM app during each of the intensive [wk 1‐12] and maintenance phases [wk 12‐24]); these data are presented descriptively by treatment arm due to low numbers. Adverse events are reported descriptively by the treatment arm.

### Ethical Considerations

The trial received multisite ethical approval from Melbourne Health (HREC/18/MH/119), which was ratified by Deakin University (2018-251), in accordance with institutional and national guidelines for research involving human participants. All participants were fully informed about the study and the voluntary nature of their involvement, including their right to withdraw at any time without penalty. Written informed consent was obtained prior to participation. Privacy and confidentiality were maintained through deidentification of collected data, which were stored securely on encrypted servers with access restricted to authorized research staff. It is not possible to identify individual participants from any images in the manuscript or supplementary material. Each participant received AUD $50 (US $35.39) shopping vouchers at the completion of baseline and 24-week assessments. Intervention group participants who used their own smartphone and/or data plan received an additional AUD $60 (US $42.46) value to reimburse data use during the intervention at the 24-week assessment.

## Results

**Figure 2.**
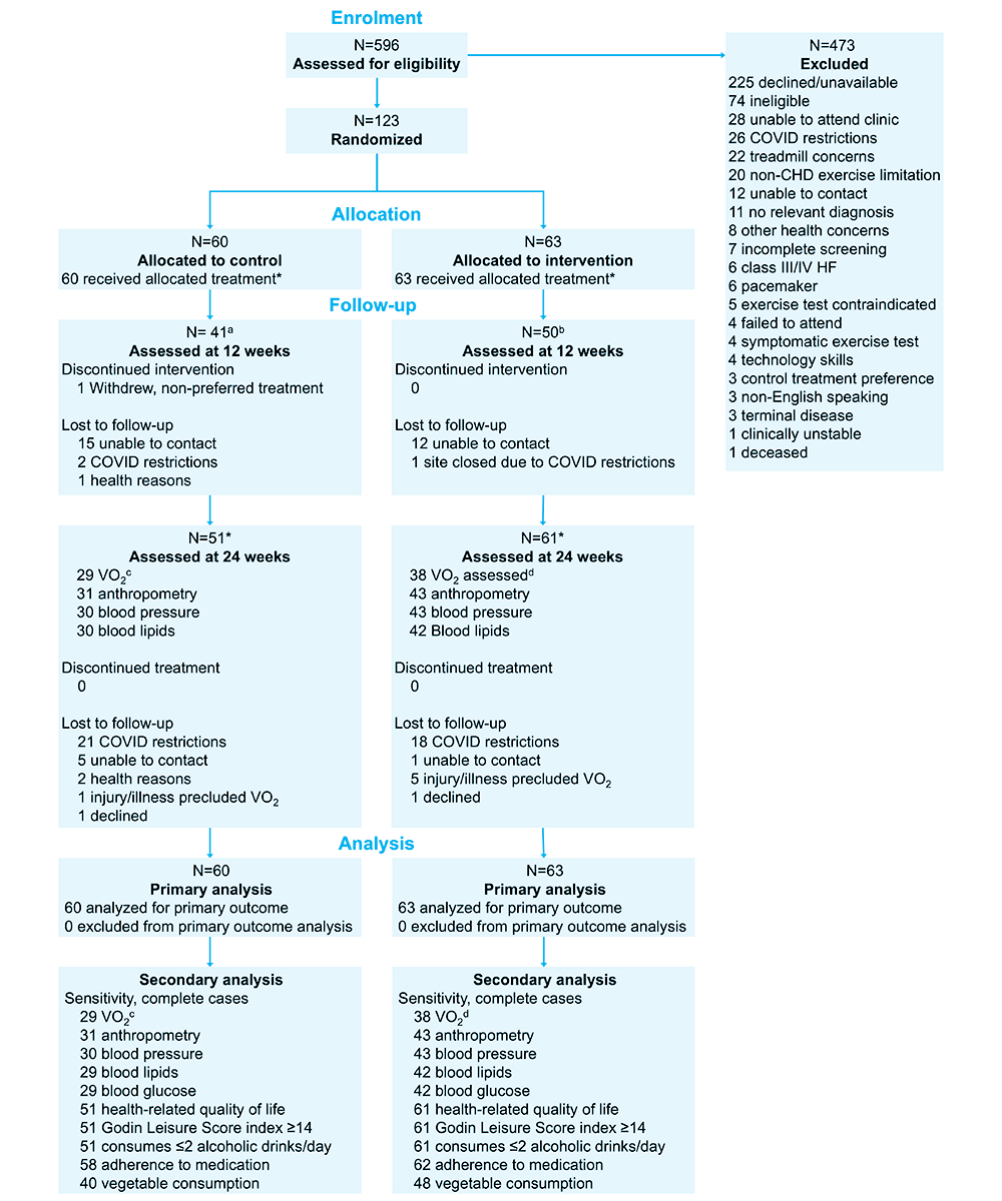
Consolidated Standards of Reporting Trials flow diagram. Randomized controlled trial, Victoria, Australia. Population: adults with coronary heart disease. *Completed lifestyle survey. ^a^n=40 for Godin leisure score index at 12 weeks; ^b^n=49 for Godin leisure score index at 12 weeks; ^c^Estimated relative VO_2_ was used when objectively measured peak relative VO_2_ was not available (measured n=27, estimated n=2); ^d^Estimated relative VO_2_ was used when objectively measured peak relative VO_2_ was not available (measured n=35, estimated n=3). CHD: coronary heart disease; HF: heart failure.

COVID-19 severely affected the recruitment and collection of objectively measured outcomes. As a result, we were unable to achieve the recruitment target. Randomization and outcome assessments completed pre- and peri-COVID were comparable between groups (Multimedia Appendix 2). Overall, 123 (55.9% of the target) participants were randomized (Figure 2); characteristics are reported in Table 2. Recruitment stopped (March 21, 2021) due to COVID-19 lockdowns and health and safety measures implemented by participating hospitals. The SCRAM intervention was delivered as planned throughout the trial. Delivery of usual care cardiac rehabilitation (control) was frequently disrupted by enforced COVID-19 restrictions, which meant that in some regions, participants may not have been offered in-person, center-based contact. We were unable to quantify the level of contact for control participants.

**Table 2.** Baseline demographic and clinical characteristics^a^.

Characteristics	Intervention (n=63)	Control (n=60)	Total (n=123)
Participants, n (%)			
Barwon Health	13 (20.6)	11 (18.3)	24 (19.5)
Western Health	12 (19)	11 (18.3)	23 (18.7)
Bendigo Health	38 (60.3)	38 (63.3)	76 (61.8)
Age (y), mean (SD)	61.3 (9.9)	60.5 (11.2)	60.9 (10.5)
Sex, n (%)			
Male	54 (85.7)	51 (85)	105 (85.4)
Female	9 (14.3)	9 (15)	18 (14.6)
Ethnicity, n (%)			
Australian	55 (87.3)	48 (80)	103 (83.7)
Other	8 (12.7)	12 (20)	20 (16.3)
Household income^b^, n (%)			
Below median	46 (73)	41 (68.3)	87 (70.7)
Above median	15 (23.8)	16 (26.7)	31 (25.2)
Do not know or refuse to answer	2 (3.2)	3 (5)	5 (4.1)
Education level, n (%)			
Bachelor degree and above	14 (22.2)	13 (22)	27 (22.1)
Less than Bachelor degree	49 (77.8)	46 (78)	95 (77.9)
Employment status, n (%)			
Employed	33 (52.4)	34 (56.6)	67 (54.5)
Unemployed	30 (47.6)	26 (43.4)	56 (45.5)
Smoking status, n (%)			
Never smoked	26 (41.3)	23 (39)	49 (40.2)
Ex-smoker	31 (49.2)	28 (47.5)	59 (48.4)
Current smoker	6 (9.5)	8 (13.6)	14 (11.5)
Medical history, n (%)			
Hypertension	30 (47.6)	35 (58.3)	65 (52.8)
BP^c^ lowering medication	47 (74.6)	41 (69.5)	88 (72.1)
Diabetes	17 (27)	12 (20.3)	29 (23.8)
Insulin^d^	4 (23.5)	6 (50)	10 (34.5)
Blood sugar-lowering medication^d^	12 (70.6)	9 (75)	21 (72.4)
High cholesterol	39 (61.9)	31 (52.5)	70 (57.4)
Cholesterol-lowering medication	54 (85.7)	52 (88.1)	106 (86.9)
Myocardial infarction	46 (73)	42 (71.2)	88 (72.1)
Angina	25 (39.7)	20 (33.9)	45 (36.9)
Stent or PCI^e^	54 (85.7)	44 (74.6)	98 (80.3)
CABG^f^	13 (20.6)	13 (22)	26 (21.3)
Atrial fibrillation	5 (7.9)	5 (8.5)	10 (8.2)
Other forms of heart disease	2 (3.2)	0 (0)	2 (1.6)

a Randomized controlled trial, Victoria, Australia. Population: adults with coronary heart disease.

b Victorian median annual household income approximately equal to AUD $90,000 (US $63,697.05) [43].

c BP: blood pressure.

d Denominators were numbers of participants with diabetes.

e PCI: percutaneous coronary intervention.

f CABG: coronary artery bypass graft.

Enforced COVID-19 restrictions meant VO_2_max could only be assessed in 67 (54.5%; intervention=38 [35 measured, 3 estimated], control=29 [27 measured, 2 estimated] participants at 24 weeks; Figure 2). Using multiple imputation, we found a between-group VO_2_max difference at 24 weeks favoring SCRAM, but it was not statistically significant (mean difference=1.61 mL/kg/min; 95% CI −1.38 to 4.61, *P*=.28).

Among secondary outcomes, SCRAM demonstrated a positive effect compared to control for diastolic blood pressure at 24 weeks (mean difference: −5.54 mm Hg; 95% CI −10.01 to ‐1.06, *P*=.02, Tables3  4). CIs included the null for between-group differences of self-reported secondary outcomes at 12 weeks (Tables5  6).

**Table 3.** Primary intention-to-treat analyses of outcomes assessed at 24 wk, using multiple imputed data^g^.

Outcome	Baseline		Wk 24		Within-group change		Between-group difference	
	Control (n=60), mean (SD)	Intervention (n=63), mean (SD)	Control (n=60), mean (SD)	Intervention (n=63), mean (SD)	Control (n=60), mean (SD)	Intervention (n=63), mean (SD)	Intervention–control (n=123), mean (95% CI)	*P* value
VO_2_max (mL/kg/min)	24.16 (8.02)	23.85 (7.77)	24.65 (7.87)	26.10 (10.72)	0.49 (8.07)	2.25 (9.92)	1.61 (–1.38 to 4.61)	.28
BP^b^ systolic (mm Hg)	138.77 (18.08)	130.85 (20.47)	135.40 (19.54)	132.23 (21.67)	–3.37 (20.79)	1.38 (22.75)	–0.10 (–6.95 to 6.76)	.98
BP diastolic (mm Hg)	80.93 (10.92)	78.64 (11.11)	84.38 (14.68)	77.97 (11.76)	3.45 (15.60)	–0.67 (12.85)	–5.54 (–10.01 to –1.06)	.02
Body mass (kg)	86.14 (15.42)	87.37 (14.80)	86.48 (12.56)	85.61 (11.11)	0.34 (12.17)	–1.76 (9.46)	–1.58 (–4.45 to 1.28)	.28
BMI (kg/m^2^)	29.47 (5.19)	29.35 (5.64)	29.59 (4.22)	28.82 (4.23)	0.12 (4.59)	–0.54 (3.09)	–0.72 (–1.79 to 0.36)	.19
Waist circumference (cm)	100.99 (12.13)	101.68 (12.60)	101.21 (11.63)	100.40 (12.25)	0.21 (10.29)	–1.28 (6.53)	–1.40 (–4.03 to 1.23)	.29
Hip circumference (cm)	101.85 (10.03)	103.27 (9.93)	101.87 (8.08)	101.55 (10.11)	0.02 (9.94)	–1.73 (5.77)	–1.30 (–3.74 to 1.14)	.29
Waist-hip ratio	0.99 (0.08)	0.98 (0.08)	1.01 (0.06)	0.99 (0.10)	0.01 (0.08)	0.00 (0.08)	–0.02 (–0.04 to 0.01)	.18
Total-C^c^ (mmol/L)	3.55 (1.02)	3.64 (1.04)	3.87 (1.52)	3.72 (1.22)	0.33 (1.48)	0.07 (0.96)	–0.22 (–0.66 to 0.22)	.32
HDL-C^d^ (mmol/L)	1.09 (0.37)	1.03 (0.28)	1.03 (0.47)	1.10 (0.40)	–0.06 (0.50)	0.06 (0.39)	0.10 (–0.06 to 0.25)	.22
LDL-C^e^ (mmol/L)	1.85 (0.92)	1.84 (0.77)	2.08 (1.35)	1.85 (1.04)	0.22 (1.46)	0.01 (0.88)	–0.22 (–0.63 to 0.20)	.31
Blood glucose (mmol/L)	6.37 (2.33)	6.22 (2.04)	6.64 (3.87)	6.32 (2.50)	0.27 (3.49)	0.10 (2.86)	–0.21 (–1.29 to 0.87)	.69
HRQoL^f^	0.78 (0.15)	0.77 (0.19)	0.79 (0.17)	0.81 (0.17)	0.01 (0.14)	0.03 (0.10)	0.01 (–0.05 to 0.07)	.73

a Randomized controlled trial, Victoria, Australia. Population: adults with coronary heart disease.

b BP: blood pressure.

c Total-C: total cholesterol.

d HDL-C: high-density lipoprotein cholesterol.

e LDL-C: low-density lipoprotein cholesterol.

f HRQoL: health-related quality of life.

**Table 4.** Primary intention-to-treat analyses of additional outcomes assessed at 24 wk, using multiple imputed data^a^.

Outcome	Baseline		Wk 24		Within-group change	Between-group difference	Within-group change	
	Control (n=60), n (%)	Intervention (n=63), n (%)	Control (n=60), n (%)	Intervention (n=63), n (%)			Odds ratio (95% CI)	*P* value
Godin LSI^b^ ≥14	45 (75)	50 (79.37)	54 (90.50)	56 (88.48)	—^c^	—	0.77 (0.20-2.91)	.70
Alcohol intake ≤2 drinks/d	54 (90)	56 (88.89)	55 (91.67)	59 (93.62)	—	—	1.35 (0.34-5.40)	.67
Medication adherence score=4	38 (63.33)	29 (46.03)	36 (59.20)	38 (60.13)	—	—	1.04 (0.50-2.17)	.91
Vegetable consumption					—	—	1.10 (0.56-2.17)	.78
≤1 serving/d	2 (3.33)	0 (0)	2 (3.33)	1 (1.75)				
1 serving/d	8 (13.33)	7 (11.11)	3 (5)	5 (7.94)				
2 servings/d	7 (11.67)	11 (17.46)	11 (18.33)	7 (11.11)				
3 servings/d	16 (26.67)	17 (26.98)	15 (25)	21 (33.33)				
4 servings/d	18 (30)	13 (20.63)	13 (21.67)	12 (19.05)				
≥5 servings/d	9 (15)	15 (23.81)	16 (26.67)	17 (26.98)				

a Randomized controlled trial, Victoria, Australia. Population: adults with coronary heart disease.

b LSI: leisure score index.

c Not available.

**Table 5.** Primary intention-to-treat analyses of health-related quality of life assessed at 12 wk, using multiple imputed data^a^.

Outcome	Baseline		12 wk		Within-group change		Between-group difference	
	Control (n=60), mean (SD)	Intervention (n=63), mean (SD)	Control (n=60), mean (SD)	Intervention (n=63), mean (SD)	Control (n=60), mean (SD)	Intervention (n=63), mean (SD)	Intervention–control (n=88), mean (95% CI)	*P* value
Health-related quality of life	0.78 (0.15)	0.77 (0.19)	0.79 (0.19)	0.78 (0.20)	0.01 (0.17)	0.01 (0.12)	–0.02 (–0.08 to 0.05)	.64

a Randomized controlled trial, Victoria, Australia. Population: adults with coronary heart disease.

**Table 6.** Primary intention-to-treat analyses of additional outcomes assessed at 12 wk, using multiple imputed data^a^.

Outcome	Baseline		12 wk		Within-group change	Between-group difference	Within-group change	
	Control (n=60), n (%)	Intervention (n=63), n (%)	Control (n=60), n (%)	Intervention (n=63), n (%)	Control (n=60), n (%)	Intervention (n=63), n (%)	Intervention–control (n=88), odds ratio (95% CI)	*P* value
Godin LSI^b^ 14	45 (75)	50 (79.37)	52 (87.37)	58 (91.30)	—^c^	—	1.51 (0.37-6.25)	.57
Alcohol intake ≤2 drinks/d	54 (90)	56 (88.89)	36 (88.67)	45 (87.81)	—	—	0.89 (0.27-3.00)	.85
Medication adherence score=4	38 (63.33)	29 (46.03)	35 (57.93)	40 (62.76)	—	—	1.23 (0.54-2.83)	.62
Vegetable consumption					—	—	1.71 (0.79-3.71)	.18
≤1 serving/d	2 (3.33)	0 (0)	2 (3.07)	1 (1.81)				
1 serving/d	8 (13.33)	7 (11.11)	9 (15.07)	9 (14.13)				
2 servings/d	7 (11.67)	11 (17.46)	6 (10.27)	7 (11.71)				
3 servings/d	16 (26.67)	17 (26.98)	23 (37.87)	13 (20.38)				
4 servings/d	18 (30)	13 (20.63)	12 (20.37)	20 (31.17)				
≥5 servings/d	9 (15)	15 (23.81)	8 (13.37)	13 (20.79)				

a Randomized controlled trial, Victoria, Australia. Population: adults with coronary heart disease.

b LSI: leisure score index.

c Not available.

Sensitivity analyses, including prognostic factors and complete case analyses, were generally consistent with the primary analyses (Multimedia Appendix 3). Per protocol analyses excluded 19 participants (control n=10, intervention n=9) for eligibility criteria violations (recent CHD diagnosis n=3, clinically stable outpatients n=15, terminal disease n=1) and 42 participants for SCRAM treatment nonadherence (Multimedia Appendix 3).

Among 22 reported adverse events (control n=6, intervention n=16), all were deemed mild or moderate, and only 1 was deemed as possibly related to treatment. There were no deaths or hospitalizations (Multimedia Appendix 4).

As part of the process evaluation, we conducted interviews with 21 trial participants who completed the study (n=11 from the intervention group and n=10 from the control group), 4/21 (19%) of whom were female participants. Participants reported high levels of usability, acceptability, and satisfaction with the SCRAM intervention. Most intervention participants (9/11, 82%) indicated that the technology was simple and easy to use. All participants agreed that SCRAM provided a flexible option for cardiac rehabilitation, allowing them to exercise in locations convenient to them while retaining expert supervision. Those who found SCRAM easy to use expressed high satisfaction with the intervention, particularly valuing the expert supervision, synchronous coaching, and activity-monitoring features.

## Discussion

### Principal Findings

This study sought to compare the effects of the SCRAM intervention on cardiorespiratory fitness, lifestyle and self-management behaviors, and HRQoL with usual cardiovascular care in adults with CHD. Overall, we found beneficial directional effects on the primary and selected secondary outcomes that favored the SCRAM intervention, but our findings are inconclusive as we were unable to recruit the a priori target sample size.

Unfortunately, the COVID-19 pandemic severely impacted this trial. We did not achieve our desired sample size, and follow-up data were lost due to enforced closures within the Australian health care services during this trial. While beneficial directional effects were generally consistent with our previous effectiveness trial [23], a lack of statistical power renders our findings, and any comparisons to previous cardiac telerehabilitation research, inconclusive. Nonetheless, it is important to report the trial to avoid publication bias [44].

Key findings were the resilience of telerehabilitation to severe cardiac rehabilitation service disruption, and its potential to deliver multifactorial supervised exercise and self-management behavioral support regardless of participants’ geographic proximity to traditional center-based cardiac rehabilitation facilities. The COVID-19 pandemic was unforeseen at the outset of our trial but significantly impacted global center-based cardiac rehabilitation delivery during the trial period, and telehealth quickly became the only safe and preferred delivery option [45-49]. Supervised exercise training was the most impacted program component globally, with approximately 76% of center-based programs reporting moderate to high impact due to COVID-19–related safety concerns [45]. While the SCRAM telerehabilitation intervention was not originally designed for such circumstances, its design enabled continuous delivery throughout COVID-19 restrictions in one of the world’s most locked down regions.

Despite these drawbacks, this trial provides insights about a number of important issues that have been highlighted for high-quality telerehabilitation. Remote exercise monitoring, such as that provided during the first phase of the SCRAM intervention, was identified as an important component of effective telerehabilitation [50] and was recently advocated as a preferred standard to optimize individualization and risk management [18]. Synchronous monitoring is desirable because it enables rehabilitation professionals to verify exercise individual responses, tolerability, and clinical stability via heart rate, electrocardiogram, and symptoms [18]. This could play an essential role in intervention effectiveness by avoiding protective risk mitigation strategies such as overly conservative exercise prescription, which has been used in the context of unsupervised home-based exercise training [51]. Indeed, rehabilitation professionals have reported synchronous monitoring could be a useful tool to mitigate adverse event risk by enabling higher quality individualization and progression of exercise training [51].

Telerehabilitation standards also highlight the use of center-based cardiopulmonary exercise testing as a gold standard component of routine pre- and postprogram assessment [18]. Our trial included cardiopulmonary exercise testing, but it was not possible for all the participants. This was related to COVID-19 during our trial but could manifest in numerous ways that contradict key aims of telerehabilitation. For example, precluding participants who are most in need of alternative delivery methods because they cannot attend clinical centers for requisite preprogram assessment. Our adapted exercise testing method removed measurement of respiratory gas exchange to manage the risk of viral transmission but was still conducted at clinical centers. A systematic review of remote exercise testing methods in cardiac rehabilitation found that this is an underexplored area, with limited evidence demonstrating the validity of the six-minute walk test [52]. While this is commonly used in many clinical settings, it does not provide comparable data to cardiopulmonary exercise testing. This highlights an urgent need for evidence-based methods for remotely measuring exercise capacity as part of preintervention and postintervention assessments.

Cardiac rehabilitation interventions should comprehensively provide all evidence-based core components, regardless of the delivery model [18]. The SCRAM intervention improved upon our earlier REMOTE-CR intervention [23] by adding multifactorial behavior change education and support. However, this was delivered via push notifications rather than more intensive teleconsultation methods that have been recommended [18], and did not provide all types of recommended support over and above exercise training [11,53,54]. The predominance of physical activity and exercise-related features is common in cardiac telerehabilitation [19]. While exercise training is a pivotal part of comprehensive cardiac rehabilitation [55], these gaps represent areas for improvement to ensure alignment with best practices in cardiac rehabilitation.

The exercise monitoring features of SCRAM were well suited for monitoring aerobic exercise performance but less capable of monitoring key aspects of strength exercise such as volume, load, and technique. Since strength exercise is also an important component of cardiac rehabilitation [55,56], this represents another area of opportunity to ensure telerehabilitation more fully aligns with best practices in cardiac rehabilitation.

### Strengths and Limitations

Our trial included a robust multicenter design, an objective primary outcome [57], a large diverse geographic area (including urban and regional sites), and an intervention that aligns with contemporary standards in cardiac telerehabilitation [18]. There were several limitations of our trial. First, despite considerable effort, COVID-19 response policies enforced at trial sites meant we could not recruit the required number of participants, which meant the study was underpowered. Also, due to enforced closures at the study sites, there was a large amount of missing follow-up data. Results need to be interpreted cautiously with these considerations in mind. Second, self-reported data for some outcomes had to be collected via telephone. Third, COVID-19 restrictions impacted access to in-person center-based cardiac rehabilitation services for control group participants, although they could access other telehealth services if offered by the trial sites. As a result, our comparator of usual care changed throughout the conduct of this trial. Ours was not the only trial affected by the COVID-19 pandemic. A recent paper highlighted the need to acknowledge its impact on study conduct, and the importance of reporting findings despite this [58].

### Implications

Uninterrupted delivery across a large geographic area throughout the COVID-19 pandemic demonstrated promising capability to reinforce and augment center-based cardiac rehabilitation by addressing barriers that contribute to suboptimal and inequitable access. Further research is needed to conclusively assess treatment effects and understand how the SCRAM telerehabilitation platform can be translated into clinical practice.

### Conclusion

This was an underpowered trial and findings were inconclusive, but SCRAM did not lead to a clinically important difference in VO_2_max compared to usual cardiac care. SCRAM was resilient to COVID-19–related disruptions that significantly impacted the delivery of cardiac rehabilitation and supervised exercise training in particular. The SCRAM telerehabilitation intervention aligns with several contemporary standards for cardiac telerehabilitation, and further research is needed to conclusively assess treatment effects and understand how cardiac telerehabilitation can be translated into routine practice to augment center-based delivery and enhance equity of access.

## Supplementary material

10.2196/66074 Multimedia Appendix 1 Statistical analysis plan.

10.2196/66074 Multimedia Appendix 2 Summary of participant recruitment during the COVID-19 period.

10.2196/66074 Multimedia Appendix 3 Sensitivity analyses.

10.2196/66074 Multimedia Appendix 4 Safety outcomes.

10.2196/66074 Checklist 1 Consolidated Standards of Reporting Trials checklist, Smartphone Cardiac Rehabilitation, Assisted Self-Management randomized controlled trial, Victoria, Australia.

## Abbreviations

CHD: coronary heart disease
CVD: cardiovascular disease
HRQoL: health-related quality of life
REDCap: Research Electronic Data Capture
REMOTE-CR: remote exercise monitoring trial for exercise-based cardiac rehabilitation
RR: risk ratio
SCRAM: Smartphone Cardiac Rehabilitation, Assisted Self-Management
VO_2_max: maximal aerobic exercise capacity

## References

[1] Global Burden of Cardiovascular Diseases and Risks 2023 Collaborators (2025). Global, regional, and national burden of cardiovascular diseases and risk factors in 204 countries and territories, 1990-2023. J Am Coll Cardiol.

[2] Laranjo L, Lanas F, Sun MC (2024). World Heart Federation roadmap for secondary prevention of cardiovascular disease: 2023 update. Glob Heart.

[3] O’Gara PT, Kushner FG, Ascheim DD (2013). 2013 ACCF/AHA guideline for the management of ST-elevation myocardial infarction: executive summary: a report of the American College of Cardiology Foundation/American Heart Association task force on practice guidelines: developed in collaboration with the American College of Emergency Physicians and Society for Cardiovascular Angiography and Interventions. Catheter Cardiovasc Interv.

[4] Levine GN, Bates ER, Blankenship JC (2011). 2011 ACCF/AHA/SCAI guideline for percutaneous coronary intervention: a report of the American College of Cardiology Foundation/American Heart Association Task Force on Practice Guidelines and the Society for Cardiovascular Angiography and Interventions. Circulation.

[5] Hillis LD, Smith PK, Anderson JL (2011). 2011 ACCF/AHA guideline for coronary artery bypass graft surgery: executive summary: a report of the American College of Cardiology Foundation/American Heart Association Task Force on Practice Guidelines. Circulation.

[6] Dibben GO, Faulkner J, Oldridge N (2023). Exercise-based cardiac rehabilitation for coronary heart disease: a meta-analysis. Eur Heart J.

[7] Balady GJ, Williams MA, Ades PA (2007). Core components of cardiac rehabilitation/secondary prevention programs: 2007 update: a scientific statement from the American Heart Association Exercise, Cardiac Rehabilitation, and Prevention Committee, the Council on Clinical Cardiology; the Councils on Cardiovascular Nursing, Epidemiology and Prevention, and Nutrition, Physical Activity, and Metabolism; and the American Association of Cardiovascular and Pulmonary Rehabilitation. Circulation.

[8] Cowie A, Buckley J, Doherty P (2019). Standards and core components for cardiovascular disease prevention and rehabilitation. Heart.

[9] Piepoli MF, Corrà U, Adamopoulos S (2014). Secondary prevention in the clinical management of patients with cardiovascular diseases. Core components, standards and outcome measures for referral and delivery: a policy statement from the cardiac rehabilitation section of the European Association for Cardiovascular Prevention & Rehabilitation. Endorsed by the Committee for Practice Guidelines of the European Society of Cardiology. Eur J Prev Cardiol.

[10] Suaya JA, Shepard DS, Normand SLT, Ades PA, Prottas J, Stason WB (2007). Use of cardiac rehabilitation by Medicare beneficiaries after myocardial infarction or coronary bypass surgery. Circulation.

[11] Woodruffe S, Neubeck L, Clark RA (2015). Australian Cardiovascular Health and Rehabilitation Association (ACRA) core components of cardiovascular disease secondary prevention and cardiac rehabilitation 2014. Heart Lung Circ.

[12] Carlson JJ, Johnson JA, Franklin BA, VanderLaan RL (2000). Program participation, exercise adherence, cardiovascular outcomes, and program cost of traditional versus modified cardiac rehabilitation. Am J Cardiol.

[13] Martin BJ, Hauer T, Arena R (2012). Cardiac rehabilitation attendance and outcomes in coronary artery disease patients. Circulation.

[14] Chindhy S, Taub PR, Lavie CJ, Shen J (2020). Current challenges in cardiac rehabilitation: strategies to overcome social factors and attendance barriers. Expert Rev Cardiovasc Ther.

[15] Clark RA, Conway A, Poulsen V, Keech W, Tirimacco R, Tideman P (2015). Alternative models of cardiac rehabilitation: a systematic review. Eur J Prev Cardiol.

[16] Platz K, Kools S, Howie-Esquivel J (2023). Benefits, facilitators, and barriers of alternative models of cardiac rehabilitation: a qualitative systematic review. J Cardiopulm Rehabil Prev.

[17] McDonagh ST, Dalal H, Moore S (2023). Home-based versus centre-based cardiac rehabilitation. Cochrane Database Syst Rev.

[18] Scherrenberg M, Falter M, Abreu A, Aktaa S, Busnatu S, Casado-Arroyo R (2025). Standards for cardiac telerehabilitation: a scientific statement of the European Association of Preventive Cardiology (EAPC) and the Association of Cardiovascular Nursing & Allied Professions (ACNAP) of the ESC, and the ESC Working Group on e-Cardiology. Eur Heart J.

[19] Golbus JR, Lopez-Jimenez F, Barac A (2023). Digital technologies in cardiac rehabilitation: a science advisory from the American Heart Association. Circulation.

[20] Thomas RJ, Petersen CE, Olson TP, Beatty AL, Ding R, Supervia M (2021). Asynchronous and synchronous delivery models for home-based cardiac rehabilitation: a scientific review. J Cardiopulm Rehabil Prev.

[21] Maddison R, Rawstorn JC, Rolleston A (2014). The remote exercise monitoring trial for exercise-based cardiac rehabilitation (REMOTE-CR): a randomised controlled trial protocol. BMC Public Health.

[22] Rawstorn JC, Gant N, Meads A, Warren I, Maddison R (2016). Remotely delivered exercise-based cardiac rehabilitation: design and content development of a novel mHealth platform. JMIR mHealth uHealth.

[23] Maddison R, Rawstorn JC, Stewart RAH (2019). Effects and costs of real-time cardiac telerehabilitation: randomised controlled non-inferiority trial. Heart.

[24] Rawstorn JC, Gant N, Rolleston A (2018). End users want alternative intervention delivery models: usability and acceptability of the REMOTE-CR exercise-based cardiac telerehabilitation program. Arch Phys Med Rehabil.

[25] (2003). The assessment and management of cardiovascular risk. https://www.tewhatuora.govt.nz/assets/Publications/Cardiovascular-Publications/assessment-and-management-of-cadriovasular-risk.pdf.

[26] Maddison R, Pfaeffli L, Whittaker R (2015). A mobile phone intervention increases physical activity in people with cardiovascular disease: results from the HEART randomized controlled trial. Eur J Prev Cardiol.

[27] Maddison R, Stewart R, Doughty R (2018). Text4Heart II - improving medication adherence in people with heart disease: a study protocol for a randomized controlled trial. Trials.

[28] Pfaeffli Dale L, Whittaker R, Jiang Y, Stewart R, Rolleston A, Maddison R (2015). Text message and internet support for coronary heart disease self-management: results from the Text4Heart randomized controlled trial. J Med Internet Res.

[29] Rawstorn JC, Ball K, Oldenburg B (2020). Smartphone Cardiac Rehabilitation, Assisted Self-Management versus usual care: protocol for a multicenter randomized controlled trial to compare effects and costs among people with coronary heart disease. JMIR Res Protoc.

[30] Begg C, Cho M, Eastwood S (1996). Improving the quality of reporting of randomized controlled trials. the CONSORT statement. JAMA.

[31] Hoffmann TC, Glasziou PP, Boutron I (2014). Better reporting of interventions: template for intervention description and replication (TIDieR) checklist and guide. BMJ.

[32] American College of Sports Medicine (2017). ACSM’s Exercise Testing and Prescription.

[33] (1997). Godin Leisure-Time Exercise Questionnaire. Med Sci Sports Exerc.

[34] Livingstone KM, McNaughton SA (2016). Diet quality is associated with obesity and hypertension in Australian adults: a cross sectional study. BMC Public Health.

[35] Australian Government, Department of Health for clarity (2013). Australian dietary guidelines. https://www.nhmrc.gov.au/sites/default/files/documents/australian-dietary-guidelines-2013.pdf.

[36] Park Y, Dodd KW, Kipnis V (2018). Comparison of self-reported dietary intakes from the Automated Self-Administered 24-h recall, 4-d food records, and food-frequency questionnaires against recovery biomarkers. Am J Clin Nutr.

[37] Subar AF, Kirkpatrick SI, Mittl B (2012). The Automated Self-Administered 24-Hour Dietary Recall (ASA24): a resource for researchers, clinicians, and educators from the National Cancer Institute. J Acad Nutr Diet.

[38] Bush K, Kivlahan DR, McDonell MB, Fihn SD, Bradley KA (1998). The AUDIT alcohol consumption questions (AUDIT-C): an effective brief screening test for problem drinking. Ambulatory Care Quality Improvement Project (ACQUIP). Alcohol Use Disorders Identification Test. Arch Intern Med.

[39] Morisky DE, Green LW, Levine DM (1986). Concurrent and predictive validity of a self-reported measure of medication adherence. Med Care.

[40] Maxwell A, Özmen M, Iezzi A, Richardson J (2016). Deriving population norms for the AQoL-6D and AQoL-8D multi-attribute utility instruments from web-based data. Qual Life Res.

[41] Gao L, Maddison R, Rawstorn J (2020). Economic evaluation protocol for a multicentre randomised controlled trial to compare Smartphone Cardiac Rehabilitation, Assisted Self-Management (SCRAM) versus usual care cardiac rehabilitation among people with coronary heart disease. BMJ Open.

[42] Borm GF, Fransen J, Lemmens WAJG (2007). A simple sample size formula for analysis of covariance in randomized clinical trials. J Clin Epidemiol.

[43] (2021). Income and work: census. Australian Bureau of Statistics.

[44] Dickersin K, Chan S, Chalmers TC, Sacks HS, Smith H (1987). Publication bias and clinical trials. Control Clin Trials.

[45] Ghisi G L de M, Xu Z, Liu X (2021). Impacts of the COVID-19 pandemic on cardiac rehabilitation delivery around the world. Glob Heart.

[46] Champion S, Clark RA, Tirimacco R, Tideman P, Gebremichael L, Beleigoli A (2022). The impact of the SARS-CoV-2 virus (COVID-19) pandemic and the rapid adoption of telehealth for cardiac rehabilitation and secondary prevention programs in rural and remote Australia: a multi-method study. Heart Lung Circ.

[47] Cartledge S, Thomas EE, Murphy B (2023). Impact of early COVID-19 waves on cardiac rehabilitation delivery in Australia: a national survey. Heart Lung Circ.

[48] McHale S, Neubeck L, Rowat A, Dawkes S, Hanson CL (2023). Understanding cardiac rehabilitation delivery in Scotland during the COVID-19 pandemic: lessons for the future. Br J Card Nurs.

[49] O’Doherty AF, Humphreys H, Dawkes S (2021). How has technology been used to deliver cardiac rehabilitation during the COVID-19 pandemic? An international cross-sectional survey of healthcare professionals conducted by the BACPR. BMJ Open.

[50] Gallegos-Rejas VM, Rawstorn JC, Gallagher R, Mahoney R, Thomas EE (2024). Key features in telehealth-delivered cardiac rehabilitation required to optimize cardiovascular health in coronary heart disease: a systematic review and realist synthesis. Eur Heart J Digit Health.

[51] Rawstorn JC, Subedi N, Koorts H (2025). Stakeholder perceptions of factors contributing to effective implementation of exercise cardiac telerehabilitation in clinical practice. Eur J Cardiovasc Nurs.

[52] Hwang R, Fan T, Bowe R (2022). Home-based and remote functional exercise testing in cardiac conditions, during the COVID-19 pandemic and beyond: a systematic review. Physiotherapy.

[53] Byrne RA, Rossello X, Coughlan JJ (2023). 2023 ESC Guidelines for the management of acute coronary syndromes. Eur Heart J.

[54] (2023). The BACPR standards and core components for cardiovascular disease prevention and rehabilitation 2023. https://www.cardiacrehabilitation.org.uk/site/docs/BACPR-Standards-and-Core-Components-2023.pdf.

[55] (2024). Correction to: 2023 ESC Guidelines for the management of acute coronary syndromes: developed by the task force on the management of acute coronary syndromes of the European Society of Cardiology (ESC). Eur Heart J Acute Cardiovasc Care.

[56] Verdicchio C, Freene N, Hollings M (2023). A clinical guide for assessment and prescription of exercise and physical activity in cardiac rehabilitation. A CSANZ position statement. Heart Lung Circ.

[57] Hannan EL (2008). Randomized clinical trials and observational studies: guidelines for assessing respective strengths and limitations. JACC Cardiovasc Interv.

[58] Perlis RH, Haneuse S, Rubenfeld GD, Fihn SD, Rivara FP (2021). Reporting clinical studies affected by the COVID-19 pandemic: guidelines for authors. JAMA Netw Open.

