# Supplementary material for: Smartphone Cardiac Rehabilitation, Assisted Self-Management (SCRAM) Versus Usual Care: Multicenter Randomized Controlled Trial
Source: JMIR Mhealth Uhealth. 2026 Mar 17;14:e66074. doi: 10.2196/66074 (PMC12994882; doi:10.2196/66074)
Supplement: Multimedia Appendix 1 [file mhealth-v14-e66074-s001.docx]

**Multimedia Appendix 2 Statistical Analysis Plan**

| **Trial Full Title** | Smartphone Cardiac Rehabilitation Assisted self-Management (SCRAM): A 21^st^ Century Approach for Improving Self-Management of Heart Disease |
| --- | --- |
| **Trial Short Title** | SCRAM |
| **Trial Registration** | ACTRN 12618001458224 |
| **Protocol Version** | 1 |
| **Chief Investigators** | Ralph Maddison, Kylie Ball, Brian Oldenberg, Clara Chow, Sarah McNaughton, Jonathan Rawstorn, Karen Lamb, Lan Gao, Voltaire Nadurata, Chris Neil, John Amereena, Marj Moodie |
| **Trial Statisticians** | Karen Lamb and Peixuan Li |
| **Sap Authors** | Karen Lamb |
| **Sap Version (date)** | 5.0 (23/01/2023) |

**SAP Revision History**

| **Version** | **Reason(s) for change** | **Date** |
| --- | --- | --- |
| 1.0 | Initial version | 06/10/2021 |
| 2.0 | Subgroup analyses confirmed and updated  Details on COVID-19 interruptions documented in the population section  Secondary outcomes updated: physical activity key secondary outcome as directly relates to intervention. Two dietary outcomes removed (DGI and discretionary food) as US survey incorrectly administered rather than Australian survey, impacting the interpretation of findings. Triglycerides removed as a secondary outcome as not collected.  Analysis section updated to include details of analysis for waist-hip ratio and vegetable consumption. | 29/06/2022 |
| 3.0 | Adherence definition confirmed and updated. | 01/08/2022 |
| 4.0 | Updated time points of data assessment in introduction to clarify that although 12 week assessments were planned (shown in Figure 1), this was only possible for self-report outcomes due to COVID-19 disruptions.  Additional tables added to include estimates for outcomes at 12-weeks for secondary outcomes with 12-week data available. | 13/09/2022 |
| 5.0 | All changes indicated here were undertaken post-unblinding. These must be acknowledged as such in the trial report/publications.   1. It was planned that Poisson regression would be used for the secondary outcome frequency of vegetable consumption. On further review, this variable was censored at an upper limit of 5 or more serves per day. Therefore, ordinal regression was used to analyse this outcome. Throughout this plan, any reference to this as a count outcome has been changed to ordinal. 2. Due to the small sample size, large number of secondary outcomes, small numbers in some categories of covariates and the volume of missing data, it was not possible to include many covariates in the multiple imputation model. In the final statistical analysis plan, it was specified that age, sex, type of diagnosis (e.g., angina, myocardial infarction, and coronary revascularisation), in addition to any other covariates that appear to be associated with missingness would be included in the multiple imputation model. This was not possible due to the issues alluded to. Therefore, multiple imputation models were limited to include only the outcomes and stratification factors sex and study site. Two separate multiple imputation models had to be conducted (separating the secondary outcomes into different models) due to the small sample size. 3. Moderation analysis was planned by sex and study site. However, there were too few participants in some categories of these strata for these analyses to be undertaken. Only descriptive analysis was considered. 4. Table templates were updated to include vegetable intake as an ordinal outcome. 5. A table was introduced to present missing data for each outcome. 6. The CONSORT diagram template was updated based on requests from the investigator team. | 23/01/2023 |

**SAP Signatures**

I give my approval for the attached SAP entitled SCRAM dated 23^rd^ January 2023.

| **Chief Investigator** | **SAP Author** |
| --- | --- |
| Ralph Maddison  Deakin University  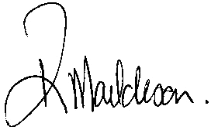  23/01/2023 | Karen Lamb  University of Melbourne  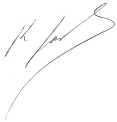  23/01/2023 |

**Table of contents**

1 Introduction 6

2 Data Source 7

3 Analysis Objectives 7

3.1 Aim 1 7

3.2 Aim 2 7

3.3 Aim 3 7

4 Analysis sets/Populations/Subgroups 7

4.1 Intention-To-Treat Population 7

4.2 Per Protocol Population 7

4.3 Safety Population 8

4.4 Subgroups 8

5 Endpoints and Covariates 8

5.1 Primary endpoint 8

5.2 Key secondary endpoint 8

5.3 Additional secondary endpoints 8

5.4 Safety endpoints 9

6 Handling of Missing Values and Other Data Conventions 9

7 Statistical Methodology 9

7.1 Statistical Procedures 9

7.1.1 Primary outcome 9

7.1.2 Secondary outcomes 9

7.1.3 Safety outcomes 10

7.2 Measures to Adjust for Multiplicity, Confounders, Heterogeneity 10

8 Sensitivity Analyses 10

9 QC Plans 10

10 Programming Plans 10

11 References 10

## 1 Introduction

A parallel two-arm randomised controlled trial was conducted to determine whether there is a difference in fitness (measured by maximal aerobic exercise capacity) at the end of the study (24 weeks) between SCRAM plus usual care (intervention) and usual care alone (control). Participants were randomised at a 1:1 ratio to receive 24 weeks of usual care alone (control) or usual care + SCRAM (intervention). Randomisation was stratified by study centre (3 study centres: Barwon, Bendigo, Western) and gender. Outcomes (all primary and secondary outcomes of interest) were to be assessed at baseline, 12-weeks (secondary time point) and 24-weeks (primary time point). However, due to COVID-19 disruptions, only self-report secondary outcomes were captured at 12-weeks. In total, the trial aimed to recruit 220 participants to randomise to intervention and control. Full study details are provided in the published study protocol ^29^.

**Figure 1 Study chart**

The following flow chart describes the timing of different aspects of the trial.

## 2 Data Source

The trial was conducted at one metropolitan (Sunshine Hospital, Western Health) and two regional (University Hospital Geelong, Barwon Health; Bendigo Hospital, Bendigo Health) health care providers in Victoria, Australia.

Eligible participants were adults (18+ years) with recently diagnosed CHD (angina, myocardial infarction, and coronary revascularisation within the previous 6 months), who were clinically stable outpatients (no CHD-related hospitalisation within 6 weeks of baseline assessment) and could understand and write English. Participants were excluded if they had New York Heart Association class III/IV heart failure, terminal disease, significant non-CHD exercise limitations, or contraindications for maximal exercise testing. Participants with an implanted

pacemaker or automated defibrillator were excluded because of wearable sensor manufacturer recommendations. Smartphone ownership was not required; participants were lent a smartphone for the duration of the intervention period if required. Participants who could not complete baseline primary outcome assessment were ineligible for randomization, and participants who experienced exercise-induced medical complications required referral for medical assessment and approval to be deemed eligible.

## 3 Analysis Objectives

### 3.1 Aim 1

The primary objective is to assess the effectiveness of SCRAM compared to usual cardiac rehabilitation care for improving fitness in adults with coronary heart disease (CHD) living in regional and rural Australia.

### 3.2 Aim 2

The secondary objective is to assess the effectiveness of SCRAM compared to usual cardiac rehabilitation care to improve self-management in adults with CHD living in regional and rural Australia. Self-management will be assessed using measurements of anthropometry, blood glucose concentration, blood pressure, composite health behaviours, alcohol consumption, dietary behaviour, health-related quality of life, medication adherence, physical activity, and exercise adherence.

### 3.3 Aim 3

To compare adverse events under both SCRAM and usual care.

Economic analyses will be conducted and reported separately to the main trial analyses. The economic analysis plan is outlined elsewhere ^30^.

## 4 Analysis sets/Populations/Subgroups

The primary analysis will be conducted according to an intention-to-treat principle, whereby the analysis will be conducted according to treatment allocation, irrespective of compliance.

The following analysis populations are planned. The status of each subject with regards to the populations and “as randomised”/“as treated” treatment group will be finalised during the blinded data review meeting.

### 4.1 Intention-To-Treat Population

This will consist of all patients who were randomised, excluding subjects who have withdrawn from the study, including use of all existing data collected to withdrawal date. Participants will be reported and analysed according to their randomised study arm (“as randomised”). This population will be used in the analysis of the efficacy study variables.

### 4.2 Per Protocol Population

This will consist of all patients who were randomised, adhered to treatment, and who did not have major protocol violations.

- A patient is defined to have adhered to treatment allocation if they followed the SCRAM intervention as planned, meaning they recorded at least 24 sessions in the SCRAM app over the 24-week intervention period, with at least 12 sessions in the first 12-week period and at least 12 in the second 12-week period.
- A major protocol violation is defined as no informed consent, violation in the inclusion or exclusion criteria, no baseline assessment available.

Patients who did not adhere to treatment or who had major protocol violation(s) will be excluded from the per protocol population. Patients will be reported and analysed according to their randomised treatment arm (“as randomised”). This population will be used in the analysis of the primary and secondary study variables.

Victoria experienced six lockdowns due to the COVID-19 pandemic during 2020 and 2021. Therefore, COVID-19 had a major impact on the SCRAM study, affecting both the ability to obtain the primary outcome and the objective secondary outcomes, in addition to affecting the usual care group who could no longer receive care in the out-patient setting. Therefore, a descriptive summary of the outcomes will be provided for patients who completed the study prior to the COVID-19 restrictions in Australia (i.e., before March 2020), those who were recruited prior to COVID-19 restrictions and partly received intervention pre-COVID-19 but completed the study during COVID-19 (since March 2020), those who were recruited since COVID-19 by treatment arm. The four periods considered in this summary will be: randomisation and both 12- and 24-week follow-up pre-COVID, randomisation and 12-week but not 24-week follow-up pre-COVID, randomisation pre-COVID but both follow-up time points peri-COVID, randomisation and both follow-up time points peri-COVID. Variations in usual care at the three trial centres since the pandemic began will be documented where details are available from the study sites.

### 4.3 Safety Population

This will consist of all patients who received at least one study treatment (including control). Patients will be reported and analysed according to their randomised study treatment (“as-randomised”). This population will be used in the analysis of adverse events.

### 4.4 Subgroups

Two subgroups will be considered: 1) sex (male/female), 2) trial centre (Western, Barwon, Bendigo). Due to difficulties obtaining the objective outcome data due to COVID-19 disruptions, the subgroup analysis will only be conducted for the key secondary outcome, physical activity.

## 5 Endpoints and Covariates

### 5.1 Primary endpoint

Maximal oxygen consumption during cardiopulmonary exercise test from at 24 weeks (VO_2_max), a continuous outcome measured in ml/kg/min. This is peak relative VO_2_ (labelled **peakrelativevo2** in database). Where this is not available for participants due to COVID disruptions to data collection, the estimated relative VO_2_ (labelled **estimatedvo2**) should be used.

### 5.2 Key secondary endpoint

Physical activity: leisure score index ≥ 14 units at 12 and 24 weeks based on the Godin Leisure Time Physical Activity Questionnaire; a binary outcome (derived variable labelled **leisure_score** in database).

### 5.3 Additional secondary endpoints

1. Average resting systolic blood pressure (mmHg) at 24 weeks; a continuous outcome (**systolicbloodpres_avg**).
2. Average resting diastolic blood pressure (mmHg) at 24 weeks; a continuous outcome (**diastolicbloodpres_avg**).
3. Body mass (kg) at 24 weeks; a continuous outcome (**weight_avg**).
4. Body mass index (kg/m^2^) at 24 weeks; a continuous outcome (**BMI**).
5. Waist circumference (cm) at 24 weeks; a continuous outcome (**waist_avg**).
6. Hip circumference (cm) at 24 weeks; a continuous outcome (**hip_avg**).
7. Waist-hip ratio at 24 weeks; a continuous outcome (**waisthipratio**).
8. Total blood lipid concentration at 24 weeks; a continuous outcome (**totalcholesterol**).
9. High-density lipoprotein concentration at 24 weeks; a continuous outcome (**hdlcholesterol**).
10. Low-density lipoprotein concentration at 24 weeks; a continuous outcome (**ldlcholesterol**).
11. Blood glucose concentration at 24 weeks; a continuous outcome (**glucose**).
12. Dietary intake: Number of portions of vegetables consumed per day at 12 and 24 weeks based; an ordinal outcome (**fv2**).
13. Alcohol consumption: consumption of ≤2 drinks/day at 12 and 24 weeks based on the 3-item Alcohol Use Disorders Identification Test-C Questionnaire; a binary outcome (**alcohol2perday**).
14. Medication adherence (Medication Adherence Scale): adherent (score=4) at 12 and 24 weeks based on the 4-item Medication Adherence Scale; a binary outcome (**adherent**).
15. Health-related quality of life (Assessment of Quality of Life 8-dimension): multi-attribute utility score based on Assessment of Quality of Life 8-dimension; a continuous outcome. (**QoL_Score**)

### 5.4 Safety endpoints

1. Self-reported changes to health status (event type, severity and relation to study). (**seeventtype**, **aeseverity**, **aerelation**)

All variables, including derived variables, are listed in Appendix 1.

## 6 Handling of Missing Values and Other Data Conventions

To describe the missing data, the frequency and percentage of participants with a missing value at baseline, 12-week post-randomisation (measurement occasion for some secondary outcomes only) and 24-weeks post-randomisation will be summarised for all study variables overall and by treatment arm (SCRAM plus usual care, usual care alone) for the intention-to-treat population. Where available, reasons for the missingness will be tabulated. In addition, baseline and demographic characteristics will be summarised overall and for those with and without a missing value at baseline, 12-week post-randomisation (measurement occasion for some secondary outcomes only) and 24-weeks post randomisation separately to examine if any characteristics appear to be associated with the presence or absence of data.

If the percentage of missing data for the primary or secondary outcomes exceeds 5%, missing outcome data will be imputed using multiple imputation. Imputation models will include participant sex and study site (stratification variables). If required due to the level of missing data, the multiple imputation analysis will be presented as the primary intention-to-treat analysis.

Primary analyses will be performed on the principle of intention-to-treat, including all patients who were randomised according to their randomised treatment arm. A per-protocol analysis will be conducted in the complete case analysis sample (see Section 4 for details of the per protocol population). Further sensitivity analyses will consider only participants who completed the study pre-COVID-19 interruptions.

## 7 Statistical Methodology

### 7.1 Statistical Procedures

#### 7.1.1 Primary outcome

The difference in mean primary outcome (VO_2_max) at 24-weeks between treatment arm will be assessed using a linear regression model, accounting for VO_2_max at baseline and stratification factors, sex and study site. Group differences will be presented with 95% confidence intervals and probability values.

#### 7.1.2 Secondary outcomes

Binary outcomes (i. leisure score index ≥ 14 units [no/yes], ii. alcohol ≤2 drinks/day [no/yes], iii. medication adherence score=4 [no/yes]) will be examined using logistic regression models fitted using generalized estimating equations to account for repeated measurements for participants. Ordinal secondary outcomes (i. vegetable consumption) will be examined using ordinal regression models with clustered standard errors to account for repeated measures. Models will include factors representing treatment arm, time and treatment arm by time interaction, as well as baseline values of each outcome and stratification factors, sex and study site.

Pre-identified potential moderators include sex and trial centre. Due to small numbers of participants in categories of each potential moderator, no formal statistical analysis is planned; only descriptive statistics within each category will be presented for the key secondary outcome (leisure score index).

Linear regression models will be fitted to examine the difference in mean continuous secondary outcomes (i. systolic blood pressure, ii. diastolic blood pressure, iii. body mass, iv. body mass index, v. waist circumference, vi. hip circumference, vii. waist-hip ratio, viii. total cholesterol, ix. HDL cholesterol, x. LDL cholesterol, xi. blood glucose concentration) at 24-weeks between treatment arm, accounting for baseline values of each outcome in respective models and stratification factors, sex and study site.

Linear mixed models will be fitted to examine differences in mean continuous secondary outcomes with two planned follow-up measurements (i. health-related quality of life score) at 12- and 24-weeks including factors representing treatment arm, time and treatment arm by time interaction, as well as baseline values of each outcome and stratification factors, sex and study site.

Standard diagnostic plots will be used to check model assumptions. Outcomes will be transformed if required.

#### 7.1.3 Safety outcomes

The number and percentage of patients with self-reported changes to health status will be documented by treatment arm.

### 7.2 Measures to Adjust for Multiplicity, Confounders, Heterogeneity

No adjustment for multiple testing will be undertaken. All secondary outcomes are exploratory and estimated effect estimates will be presented with corresponding 95% confidence intervals and probability values specified to three decimal places. Conclusions will not be based on p<0.05.

## 8 Sensitivity Analyses

In sensitivity analyses, linear regression models assessing the difference in the primary outcome (VO_2_max) will further adjust for baseline prognostic factors, age and employment status. If multiple imputation is used for primary analyses, a complete case analysis will be undertaken in sensitivity analysis.

## 9 QC Plans

Data cleaning and preparation will be undertaken by the study project manager, Emily Scanlan. Further checks will be undertaken by the primary SCRAM biostatistician (KL) and MISCH supporting biostatistician (Peixuan Li). The statistical analysis plan was completed on blinded data, without knowledge of treatment allocation.

## 10 Programming Plans

Stata version 17 will be used. A list of all tables, figures, listings and their templates can be found in Appendix 2.
