# Supplementary material for: Smartphone Cardiac Rehabilitation, Assisted Self-Management (SCRAM) Versus Usual Care: Multicenter Randomized Controlled Trial
Source: JMIR Mhealth Uhealth. 2026 Mar 17;14:e66074. doi: 10.2196/66074 (PMC12994882; doi:10.2196/66074)
Supplement: Multimedia Appendix 2 [file mhealth-v14-e66074-s002.docx]

**Multimedia Appendix 3 Summary of participant recruitment during the COVID period**

| **Time Period** | **Control** | **Intervention** |
| --- | --- | --- |
|  | **N = 60** | **N = 63** |
| Randomisation, 12- and 24-week follow-up pre-COVID | 29 (48.3%) | 33 (52.4%) |
| Randomisation and 12-week follow-up only pre-COVID | 10 (16.7%) | 7 (11.1%) |
| Randomisation only pre-COVID | 8 (13.3%) | 9 (14.3%) |
| All measures peri-COVID | 13 (21.7%) | 14 (22.2%) |
