# Supplementary material for: Smartphone Cardiac Rehabilitation, Assisted Self-Management (SCRAM) Versus Usual Care: Multicenter Randomized Controlled Trial
Source: JMIR Mhealth Uhealth. 2026 Mar 17;14:e66074. doi: 10.2196/66074 (PMC12994882; doi:10.2196/66074)
Supplement: Multimedia Appendix 3 [file mhealth-v14-e66074-s003.docx]

**Multimedia Appendix 4 Sensitivity analyses**

**Summary measures and estimated between-group differences at 24 weeks using complete case data (intention-to-treat).**

| **Outcome** | **Baseline** | | **Week 24** | | **Within-group change** | | **Between-group difference** | |
| --- | --- | --- | --- | --- | --- | --- | --- | --- |
|  | **Control** | **Intervention** | **Control** | **Intervention** | **Control** | **Intervention** | **Intervention - control** | |
|  | **Mean (SD)** | **Mean (SD)** | **Mean (SD)** | **Mean (SD)** | **Mean (SD)** | **Mean (SD)** | **Mean (95% CI)** | ***P*** |
| VO_2_ max (ml/kg/min) | 24.20 (5.91) | 25.98 (7.69) | 24.50 (6.16) | 26.46 (8.45) | 0.29 (3.36) | 0.48 (5.25) | 0.57 (-1.76, 2.89) | 0.63 |
| BP systolic (mmHg) | 140.28 (16.60) | 131.30 (17.68) | 137.63 (14.99) | 134.05 (18.63) | -2.65 (13.57) | 2.74 (15.20) | 2.58 (-3.67, 8.83) | 0.41 |
| BP diastolic (mmHg) | 81.30 (10.58) | 79.58 (11.01) | 83.72 (9.95) | 77.85 (9.73) | 2.42 (9.17) | -1.73 (10.24) | -5.18 (-9.06, -1.31) | 0.01 |
| Body mass (kg) | 88.44 (12.84) | 85.64 (12.56) | 88.42 (13.67) | 85.96 (11.66) | -0.02 (2.94) | 0.32 (2.88) | 0.40 (-0.95, 1.76) | 0.55 |
| BMI (kg/m^2^) | 29.33 (3.97) | 28.77 (5.03) | 29.36 (4.17) | 28.92 (4.70) | 0.03 (0.96) | 0.15 (0.90) | 0.15 (-0.28, 0.58) | 0.49 |
| Waist (cm) | 102.14 (9.42) | 99.88 (11.48) | 102.93 (9.42) | 99.34 (11.31) | 0.79 (3.36) | -0.55 (3.41) | -1.15 (-2.44, 0.15) | 0.08 |
| Hip (cm) | 100.09 (6.81) | 102.18 (10.25) | 101.51 (6.08) | 100.95 (10.79) | 1.42 (5.55) | -1.23 (3.36) | -2.45 (-4.39, -0.51) | 0.01 |
| Waist-hip ratio | 1.02 (0.08) | 0.98 (0.07) | 1.01 (0.05) | 0.99 (0.08) | -0.01 (0.07) | 0.01 (0.05) | 0.01 (-0.02, 0.03) | 0.54 |
| Total-C (mmol/L) | 3.59 (1.04) | 3.65 (1.06) | 3.79 (1.13) | 3.75 (1.06) | 0.20 (0.62) | 0.10 (0.56) | -0.11 (-0.39, 0.16) | 0.43 |
| HDL-C (mmol/L) | 0.98 (0.29) | 1.08 (0.27) | 1.03 (0.31) | 1.10 (0.31) | 0.05 (0.23) | 0.03 (0.26) | -0.01 (-0.13, 0.11) | 0.87 |
| LDL-C (mmol/L) | 2.06 (1.12) | 1.91 (0.81) | 2.06 (0.97) | 1.95 (0.94) | -0.00 (1.03) | 0.04 (0.52) | -0.02 (-0.42, 0.37) | 0.90 |
| Blood glucose (mmol/L) | 6.25 (2.67) | 5.98 (1.71) | 6.64 (3.30) | 6.27 (2.15) | 0.39 (1.57) | 0.29 (2.09) | -0.05 (-0.95, 0.86) | 0.92 |
| HRQoL | 0.78 (0.15) | 0.77 (0.19) | 0.79 (0.15) | 0.81 (0.16) | 0.01 (0.10) | 0.03 (0.09) | 0.01 (-0.05, 0.07) | 0.67 |
|  | **n (%)** | **n (%)** | **n (%)** | **n (%)** |  |  | **Odds ratio (95% CI)** | ***P*** |
| Godin LSI ≥14 | 45 (75.00) | 50 (79.37) | 47 (92.16) | 54 (88.52) | - | - | 0.73 (0.21, 2.51) | 0.62 |
| Alcohol intake ≤2 drinks/day | 54 (90.00) | 56 (88.89) | 46 (90.20) | 57 (93.44) | - | - | 1.35 (0.34, 5.40) | 0.67 |
| Medication adherence score=4 | 38 (63.33) | 29 (46.03) | 34 (58.62) | 37 (59.68) | - | - | 1.07 (0.51, 2.23) | 0.86 |
| Vegetable consumption |  |  |  |  | - | - | 0.79 (0.36, 1.72) | 0.55 |
| ≤1 serves/day | 1 (2.50) | 0 (0.00) | 0 (0.00) | 1 (2.08) | - | - | - | - |
| 1 serve/day | 4 (10.00) | 6 (12.50) | 2 (5.00) | 4 (8.33) | - | - | - | - |
| 2 serves/day | 3 (7.50) | 6 (12.50) | 5 (12.50) | 4 (8.33) | - | - | - | - |
| 3 serves/day | 10 (25.00) | 14 (29.17) | 10 (25.00) | 18 (37.50) | - | - | - | - |
| 4 serves/day | 16 (40.00) | 10 (20.83) | 10 (25.00) | 7 (14.58) | - | - | - | - |
| ≥5serves/day | 6 (15.00) | 12 (25.00) | 13 (32.50) | 14 (29.17) | - | - | - | - |

SD, standard deviation. CI, confidence interval. BP, blood pressure. BMI, body mass index. Total-C, total cholesterol. HDL-C, high-density lipoprotein cholesterol. LDL-C, low-density lipoprotein cholesterol. HRQoL, health-related quality of life. LSI, leisure score index. Sensitivity analysis included adjustment for stratification variables (sex, study site). Complete case sample sizes at 24 weeks varied across outcomes: VO_2_max: control n=29, intervention n=38; Systolic and diastolic blood pressure: control n=30, intervention n=43; body mass and body mass index: control n=31, intervention n=43; waist and hip circumferences and waist-hip ratio: control n=31 control, intervention n=42; total cholesterol: control n=29, intervention n=40; HDL cholesterol: control n=29, intervention n=42; LDL cholesterol: control n=26, intervention n=33; blood glucose: control n=29, intervention n=42. Health-related quality of life: control n=51, intervention n=61. Godin LSI ≥14: control n=51, intervention n=61. Alcohol consumption: control n=51, intervention n=61. Medication adherence: control n=58, intervention n=62. Vegetable consumption: control n=40, intervention n=48.

**Summary measures and estimated between-group differences at 12 weeks using complete case data (intention-to-treat).**

| **Outcome** | **Baseline** | | **12 weeks** | | **Within-group change** | | **Between-group difference** | |
| --- | --- | --- | --- | --- | --- | --- | --- | --- |
|  | **Control**  **(n=60)** | **Intervention**  **(n=63)** | **Control**  **(n=60)** | **Intervention**  **(n=63)** | **Control**  **(n=60)** | **Intervention**  **(n=63)** | **Intervention – control** | |
|  | **Mean (SD)** | **Mean (SD)** | **Mean (SD)** | **Mean (SD)** | **Mean (SD)** | **Mean (SD)** | **Mean (95% CI)** | ***P*** |
| Health-related quality of life | 0.78 (0.15) | 0.77 (0.19) | 0.80 (0.15) | 0.78 (0.19) | 0.00 (0.09) | 0.00 (0.09) | 0.00 (-0.06, 0.06) | 0.94 |
|  | **n (%)** | **n (%)** | **n (%)** | **n (%)** |  |  | **Odds ratio (95%CI)** | ***P*** |
| Godin LSI ≥14 | 45 (75.00) | 50 (79.37) | 35 (87.50) | 45 (91.84) | - | - | 1.57 (0.40, 6.09) | 0.52 |
| Alcohol intake ≤2 drinks/day | 54 (90.00) | 56 (88.89) | 35 (85.37) | 45 (90.00) | - | - | 0.89 (0.27, 2.94) | 0.85 |
| Medication adherence score=4 | 38 (63.33) | 29 (46.03) | 23 (56.10) | 32 (64.00) | - | - | 1.19 (0.52, 2.71) | 0.68 |
| Vegetable consumption |  |  |  |  |  |  | 1.70 (0.84, 3.47) | 0.14 |
| ≤1 serves/day | 1 (2.50) | 0 (0.00) | 1 (2.50) | 0 (0.00) | - | - | - | - |
| 1 serve/day | 4 (10.00) | 6 (12.50) | 4 (10.00) | 6 (12.50) | - | - | - | - |
| 2 serves/day | 3 (7.50) | 6 (12.50) | 4 (10.00) | 6 (12.50) | - | - | - | - |
| 3 serves/day | 10 (25.00) | 14 (29.17) | 16 (40.00) | 10 (20.83) | - | - | - | - |
| 4 serves/day | 16 (40.00) | 10 (20.83) | 9 (22.50) | 16 (33.33) | - | - | - | - |
| ≥5serves/day | 6 (15.00) | 12 (25.00) | 6 (15.00) | 10 (20.83) | - | - | - | - |

SD, standard deviation. CI, confidence interval. LSI, leisure score index. Sensitivity analysis included adjustment for stratification variables (sex, study site). Complete case sample sizes at 24 weeks varied across outcomes: Health-related quality of life: control n=41, intervention n=50. Godin LSI: control n=40, intervention n=49. Alcohol consumption: control n=41, intervention n=50. Medication adherence: control n=41, intervention n=50. Vegetable consumption: control n=40, intervention n=48.

**Summary measures at 24 weeks using complete case data from the per protocol sample.**

| **Outcome** | **Baseline** | | **24 weeks** | | **Within-group change** | |
| --- | --- | --- | --- | --- | --- | --- |
|  | **Control**  **(n=50)** | **Intervention**  **(n=16)** | **Control**  **(n=48)** | **Intervention**  **(n=15)** | **Control**  **(n=41)** | **Intervention**  **(n=15)** |
|  | **mean (SD)** | **mean (SD)** | **mean (SD)** | **mean (SD)** | **mean (SD)** | **mean (SD)** |
| VO_2_ max (ml/kg/min) | 25.3 (7.9) | 24.2 (7.7) | 24.6 (6.4) | 24.4 (7.6) | -0.1 (3.5) | 0.7 (4.7) |
| BP systolic (mmHg) | 137.9 (18.2) | 128.0 (22.2) | 136.2 (15.7) | 136.5 (19.2) | -3.9 (13.9) | 8.0 (15.8) |
| BP diastolic (mmHg) | 81.0 (11.3) | 81.6 (14.5) | 84.8 (9.8) | 80.3 (9.4) | 3.0 (9.5) | -2.5 (14.3) |
| Body mass (kg) | 85.1 (13.1) | 85.1 (14.1) | 88.1 (12.2) | 85.7 (13.3) | -0.1 (3.0) | 0.0 (3.2) |
| BMI (kg/m^2^) | 28.9 (3.9) | 29.4 (6.6) | 29.1 (3.5) | 29.8 (6.4) | 0.0 (1.0) | 0.0 (1.0) |
| Waist circumference (cm) | 100.0 (11.1) | 98.8 (12.0) | 102.0 (8.4) | 98.0 (11.2) | 0.9 (3.6) | -1.4 (2.6) |
| Hip circumference (cm) | 100.8 (8.4) | 102.7 (14.2) | 101.1 (6.0) | 101.0 (14.6) | 1.6 (6.1) | -1.3 (3.1) |
| Waist-hip ratio | 1.0 (0.1) | 1.0 (0.1) | 1.0 (0.0) | 1.0 (0.1) | 0.0 (0.1) | 0.0 (0.0) |
| Total-C (mmmol/L) | 3.5 (0.9) | 3.6 (0.9) | 4.0 (1.2) | 3.8 (1.2) | 0.2 (0.7) | 0.1 (0.5) |
| HDL-C (mmol/L) | 1.1 (0.4) | 1.1 (0.3) | 1.0 (0.3) | 1.2 (0.3) | 0.1 (0.2) | 0.0 (0.2) |
| LDL-C (mmol/L) | 1.8 (0.9) | 1.9 (0.9) | 2.1 (1.0) | 1.9 (1.0) | -0.1 (1.0) | 0.1 (0.4) |
| Blood glucose (mmol/L) | 6.5 (2.5) | 5.7 (1.0) | 6.8 (3.6) | 6.2 (1.7) | 0.6 (1.7) | 0.5 (1.4) |
| HRQoL | 0.8 (0.1) | 0.8 (0.2) | 0.8 (0.1) | 0.8 (0.1) | 0.0 (0.1) | 0.0 (0.1) |
|  | **n (%)** | **n (%)** | **n (%)** | **n (%)** |  |  |
| Godin LSI ≥14 | 39 (78.0) | 14 (87.5) | 39 (95.1) | 15 (100.0) | - | - |
| Alcohol intake ≤2 drinks/day | 45 (90.0) | 16 (100.0) | 37 (90.2) | 15 (100.0) | - | - |
| Medication adherence score=4 | 32 (64.0) | 8 (50.0) | 29/48 (60.4) | 10 (66.7) | - | - |
| Vegetable consumption |  |  |  |  |  |  |
| ≤1 serves/day | 2 (4.0) | 0 (0.0) | 1 (2.4) | 0 (0.0) | - | - |
| 1 serve/day | 8 (16.0) | 3 (18.8) | 2 (4.9) | 2 (13.3) | - | - |
| 2 serves/day | 6 (12.0) | 3 (18.8) | 6 (14.6) | 1 (6.7) | - | - |
| 3 serves/day | 12 (24.0) | 3 (18.8) | 9 (22.0) | 4 (26.7) | - | - |
| 4 serves/day | 14 (28.0) | 2 (12.5) | 9 (22.0) | 4 (26.7) | - | - |
| ≥5serves/day | 8 (16.0) | 5 (31.2) | 14 (34.1) | 4 (26.7) | - | - |

SD, standard deviation. CI, confidence interval. BP, blood pressure. BMI, body mass index. Total-C, total cholesterol. HDL-C, high-density lipoprotein cholesterol. LDL-C, low-density lipoprotein cholesterol. HRQoL, health-related quality of life. LSI, leisure score index.

The per protocol sample excludes participants with major protocol violations (control n=10, intervention n=9) related to eligibility criteria (recent CHD diagnosis n=3, clinically stable outpatients n=15, terminal disease n=1), and who were non-adherence to the SCRAM intervention (n=42), defined as recording <12 exercise sessions via the SCRAM app during each of the intensive (weeks 1–12) and maintenance phases (weeks 12–24).
