# Supplementary material for: Smartphone Cardiac Rehabilitation, Assisted Self-Management (SCRAM) Versus Usual Care: Multicenter Randomized Controlled Trial
Source: JMIR Mhealth Uhealth. 2026 Mar 17;14:e66074. doi: 10.2196/66074 (PMC12994882; doi:10.2196/66074)
Supplement: Multimedia Appendix 4 [file mhealth-v14-e66074-s004.docx]

**Multimedia Appendix 5 Safety outcomes**

| **Characteristic** | **Intervention** | |  | **Control** | |
| --- | --- | --- | --- | --- | --- |
|  | **Week 12** | **Week 24** |  | **Week 12** | **Week 24** |
| Total events | 5 (7.9%) | 11 (17.5%) |  | 3 (5.0%) | 3 (5.0%) |
| Event classification^b^ |  |  |  |  |  |
| Non-serious event | 4 (80.0%) | 9 (81.8%) |  | 3 (100.0%) | 2 (66.7%) |
| Death | 0 (0.0%) | 0 (0.0%) |  | 0 (0.0%) | 0 (0.0%) |
| Life-threatening | 0 (0.0%) | 0 (0.0%) |  | 0 (0.0%) | 0 (0.0%) |
| Hospitalisation | 0 (0.0%) | 0 (0.0%) |  | 0 (0.0%) | 0 (0.0%) |
| Persistent incapacity | 0 (0.0%) | 0 (0.0%) |  | 0 (0.0%) | 0 (0.0%) |
| Congenital abnormality | 0 (0.0%) | 0 (0.0%) |  | 0 (0.0%) | 0 (0.0%) |
| Medically important event | 1 (20.0%) | 2 (18.2%) |  | 0 (0.0%) | 1 (33.3%) |
| Severity^b^ |  |  |  |  |  |
| Mild | 3 (60.0%) | 3 (27.3%) |  | 2 (66.7%) | 2 (66.7%) |
| Moderate | 2 (40.0%) | 8 (72.7%) |  | 1 (33.3%) | 1 (33.3%) |
| Severe | 0 (0.0%) | 0 (0.0%) |  | 0 (0.0%) | 0 (0.0%) |
| Relationship to treatment^b^ |  |  |  |  |  |
| Unrelated | 3 (60.0%) | 8 (72.7%) |  | 3 (100.0%) | 3 (100.0%) |
| Possible | 2 (40.0%) | 2 (18.2%) |  | 0 (0.0%) | 0 (0.0%) |
| Probably | 0 (0.0%) | 1 (9.1%) |  | 0 (0.0%) | 0 (0.0%) |
| Definite | 0 (0.0%) | 0 (0.0%) |  | 0 (0.0%) | 0 (0.0%) |

^a^The total number of participants in each treatment group were used as denominators to calculate the % for total events (weeks 12 and 14: intervention n=63, control n=60).

^b^Severity and relationship to treatment data were only collected from participants who had safety events. These numbers were used as denominators to calculate the % (week 12: intervention n=5, control n=3; week 24: intervention n=11, control n=3).
